# Maize EMBRYO SAC family peptides interact differentially with pollen tubes and fungal cells

**DOI:** 10.1093/jxb/erv268

**Published:** 2015-06-12

**Authors:** Mayada Woriedh, Rainer Merkl, Thomas Dresselhaus

**Affiliations:** ^1^Cell Biology and Plant Biochemistry, Biochemie-Zentrum Regensburg, University of Regensburg, 93053 Regensburg, Germany; ^2^Institute of Biophysics and Physical Biochemistry, Biochemie-Zentrum Regensburg, University of Regensburg, 93053 Regensburg, Germany

**Keywords:** Cysteine rich protein (CRP), defensin, *Fusarium graminearum*, maize, pollen tube, ROS, *Ustilago maydis.*

## Abstract

EMBRYO SAC1-4 (ES1-4) peptides belong to the defensin subgroup of cysteine-rich peptides known to mediate pollen tube burst in *Zea mays* (maize). ES1-4 are reported here to also be capable of inhibiting germination and growth of the maize fungal pathogens *Fusarium graminearum* and *Ustilago maydis* at higher concentrations. Dividing the peptides into smaller pieces showed that a 15-amino-acid peptide located in a highly variable loop region lacking similarity to other defensins or defensin-like peptides binds to maize pollen tube surfaces, causing swelling prior to burst. This peptide fragment and a second conserved neighbouring fragment showed suppression of fungal germination and growth. The two peptides caused swelling of fungal cells, production of reactive oxygen species, and finally the formation of big vacuoles prior to burst at high peptide concentration. Furthermore, peptide fragments were found to bind differently to fungal cells. In necrotrophic *F. graminearum,* a peptide fragment named ES-d bound only at cell surfaces whereas the peptide ES-c bound at cell surfaces and also accumulated inside cells. Conversely, in biotrophic *U. maydis*, both peptide fragments accumulated inside cells, but, if applied at higher concentration, ES-c but not ES-d accumulated mainly in vacuoles. Mapping of peptide interaction sites identified amino acids differing in pollen tube burst and fungal response reactions. In summary, these findings indicate that residues targeting pollen tube burst in maize are specific to the ES family, while residues targeting fungal growth are conserved within defensins and defensin-like peptides.

## Highlight

EMBRYO SAC family peptides show multiple activities in mediating pollen tube burst and fungal growth inhibition. They interact differently with cell surface receptors of pollen tube and fungal cells.

## Introduction

The *EMBRYO SAC* (*ES*) gene family of maize consists of four members, *ES1* to *ES4*, which are highly similar to each other and encode small cysteine-rich peptide precursors of 91 to 92 amino acids length (Cordts *et al.*, 2001). All peptides possess a conserved signal peptide and their structure is typical for defensins, in which cysteine bridges stabilize an αβ-motif (CSαβ) consisting of an α-helix and a triple-stranded antiparallel β-sheet (Lacerda *et al.*, 2014). Mature ES peptides of 61-amino-acid length appear to fold through four disulfide bridges (Cordts *et al.*, 2001; Amien *et al.*, 2010). Defensins are only found in eukaryotes. In plants, they include lectins/antimicrobial peptides, proteinase/amylase inhibitors, and γ-thionins. They are sometimes also described as knottins or cysteine-knot peptides of small inhibitors, toxins, and lectins (Gelly *et al.*, 2004; Marchler-Bauer *et al.*, 2015). Defensins in metazoan species, mainly from the arthropoda, include antibacterial defensins and scorpion α-neurotoxins (Smith *et al.*, 2013), but also α-amylase inhibitors, antifungal proteins, and proteins annotated as proteinase inhibitors (Schultz *et al.*, 1998; Letunic *et al.*, 2015). Plant defensins have previously been shown to be required as part of the innate immune system to protect organs such as roots, flowers, fruits, and seeds from pathogen attack (e.g. Iwai *et al.*, 2002; Dracatos *et al.*, 2014; Ji *et al.*, 2015).

Recent studies have demonstrated that plant defensins and defensin-like proteins (DEFL) also act as key signalling molecules during reproductive processes, especially along the pollen tube journey (Dresselhaus and Franklin-Tong, 2013; Qu et al., 2015). As polymorphic small secreted peptides, they are involved, for example, in signalling during pollen–pistil interactions (SCR/SP11: Schopfer *et al.*, 1999), in pollen-tube guidance (LUREs: Okuda *et al.*, 2009; Goto *et al.*, 2011; Takeuchi and Higashiyama, 2012), and in sperm cell release (ES4: Amien *et al.*, 2010). The *ES* gene family was previously shown to be highly expressed in the cells of the egg apparatus of maize. After fertilization, the genes were switched off and expression was not detectable during seed development or in vegetative tissues of maize (Cordts *et al.*, 2001; Amien *et al.*, 2010). ES4 was further studied and shown to mediate pollen tube burst via opening of the potassium channel KZM1 (Amien *et al.*, 2010). In contrast, scorpion toxins related to ES peptides appear to act at multiple ion channels and were shown to block the pore of potassium channels (Quintero-Hernandez *et al.*, 2013).

Considering that the various subclasses of defensins mainly act either as antimicrobial peptides or as toxins against higher organisms, the aim of this study was to elucidate the various functional activities and specificity of the whole ES1–4 peptide family, both in reproductive processes and defence reactions. Thus, the activity of various ES peptides and their mutant versions on pollen tube burst was investigated. The biotrophic fungus *Ustilago maydis* and the necrotrophic fungus *Fusarium graminearum* served as microbial targets to quantitatively study the effect of these peptides on fungal growth behaviour and response(s). Investigations were restricted to fungi because it is known that ES4 does not inhibit growth of bacterial pathogens (Amien *et al.*, 2010). Moreover, important peptide fragments and individual residues within ES1–4 involved in the interactions with their pollen tube and fungal receptors were identified and characterized.

## Materials and methods

### Growth, culture, and harvest of plant and fungal material

Maize plants of inbred line A188 were grown under standard greenhouse conditions at 26°C with 16h light (22°C in the dark) and a relative air humidity of about 60%. Pollen was collected at anthesis (stage R1; stages after Abendroth *et al.*, 2011) on paper sheets after tassels had been shaken softly the evening before to remove old pollen grains. Conidiation of *F. graminearum* strain ph1 was induced on synthetic nutrient-poor medium on plates incubated at 18°C under near-UV white light condition after Woriedh *et al.* (2011). Sporulation of *U. maydis* strain sg200 was induced in liquid complete medium and spores were incubated on a rotor at 28°C in the dark. *F. graminearum* conidia and *U. maydis* spores were collected and washed with sterile deionized water. Aliquots with 10% glycerol were kept at −80°C at OD_600_ of 1 for further studies. *F. graminearum* and *U. maydis* were cultured in pollen germination medium [PGM: 0.0005% H_3_BO_3_, 10mM CaCl_2_, 0.05mM KH_2_PO_4_, 10% sucrose, and 6% PEG 4000 (Schreiber *et al.*, 2004)].

### Peptide synthesis and dilution

Predicted mature peptides of ES1 (gi|162459879), ES4 (gi|162461029), mutated ES4, as well as peptides ESa–e, mutated peptides, and labelled peptides were chemically synthesized in acetonitrile with an HPLC-purity of 95% (Biomatik, Canada). Peptides were dissolved in DMSO and thereafter diluted with PGM prior to use according to Amien *et al.* (2010) and Woriedh *et al.* (2013). DMSO concentration to PGM never exceeded 2%. ES4 (61 amino acids) was labelled using the fluorescent dye Rhodamine (Sigma) according to the instruction manual (AAT Bioquest, Inc.).

### Pollen tube burst assay

The assay was applied as described (Woriedh *et al.*, 2013). Briefly, freshly collected pollen was immediately distributed onto Petri dishes containing droplets of 10 µL PGM at different pH values. Pollen tubes started to burst or to grow after about 10min. Pollen tubes were subjected to further studies only if germination rates exceeded 80%. After optimization, PGM at pH 5 was applied to all pollen tube assays. A 10 µL solution of the above peptides or acetonitrile as a negative control was added to each droplet of germinated pollen using a Cell-TramH Air pump (Eppendorf) and a glass micropipette. During this assay, droplets were observed using an Eclipse TE2000 inverted microscope (Nikon) equipped with a 1.4 Megapixel digital AxioCam MRm camera (black and white) and AxioVision digital image processing software (Release 4.8).

### Fungal germination inhibitory assay

Examination of the inhibitory effect of peptides on germination of *F. graminearum* conidia and *U. maydis* spores was performed in 96-well microtitre plates. A 3 µL solution of peptides or control PGM was added to 96 µL PGM supplemented with 1 µL (OD_600_ = 1) of *F. graminearum* conidia or *U. maydis* spores each expressing 3xGFP marker protein. Microtitre plates were incubated at 28°C at 150rpm in the dark and measured using spectrophotometry by relative absorbance at 595nm using a Multiskan GO spectrophotometer (Thermo Scientific). Inhibition studies of *F. graminearum–*3xGFP and *U. maydis–*3xGFP in culture were controlled microscopically using an Eclipse TE2000 inverted microscope (Nikon) as described above. GFP was excited with UV light using a HC Alexa488/EGFP filter (F36-525, EX: 472/30, DM: 495, BA: 520/35 BP).

### Peptide binding assays

For maize pollen tubes, maize pollen was germinated on a cellulose acetate membrane (Sigma: dialyser capacity 10–200 μL, MWCO 500Da, diameter 11mm) and flooded with a droplet of 10 µL PGM. Cellulose acetate membranes were washed three times with PGM before use. Around 10min after germination of pollen tubes, 10 µL of TAMRA-ES-a, Dabcyl-ES-c, TAMRA-ES-d, or Rhodamine-ES4 was applied to the cellulose membrane, gently washed with PGM, and immediately visualized under bright field and fluorescence using an Aoptome.2 microscope (Zeiss) equipped with an Axio camera 503 mono and an Axio camera 105 colour. The ZEN 2012 SP2 digital image software (Blue edition) was used to capture and process images. TAMRA and rhodamine were excited with UV light using filter set 43 HE, 489047–9901 (EX: BP 550/25, Beamsplitter: FT 570, EM: BP 605/70). Dabcyl was excited with UV light using filter set 49, 488049–9901 (EX: G 365, Beamsplitter: FT 395, EM: BP 445/50).

For *F. graminearum* conidia or *U. maydis* spores, a 6 µL solution of TAMRA-ES-a, Dabcyl-ES-c, TAMRA-ES-d, or Rhodamine-ES4 was added to 192 µL PGM supplemented with 2 µL (OD_600_ = 1) of conidia or spores at 28°C in the dark. Cells were germinated for 3, 6, 12, and 24h, and then washed with PGM before visualization on a cellulose acetate membrane using an Aoptome.2 microscope (Zeiss) as described above. Cellulose acetate membranes were washed three times with PGM before usage.

For *F. graminearum* wild-type mycelia, 2 µL (OD_600_ = 1) of conidia were germinated in 192 µL PGM at 28°C in the dark for 24h, and then incubated with 6 µL of labelled peptides for 3, 6, 12, and 24h before visualization as described above.

### Histochemical detection of reactive oxygen species

Production of reactive oxygen species (ROS) was detected by staining of the superoxide (O_2_^-^) anion with nitrotetrazolium blue chloride (NBT; Sigma) according to Semighini and Harris (2008); 2.5mM NBT (Sigma) was dissolved in 5mM Mops. To stain wild-type *F. graminearum* conidia or *U. maydis* spores, a 6 µL solution of unlabelled peptides was added to 192 µL PGM supplemented with 2 µL (OD_600_ = 1) of conidia or spores at 28°C in the dark, germinated for 24h, and then incubated again under the same conditions with 200 µL NBT solution for 2h. Samples were washed with PGM before visualization on a cellulose membrane under bright field using an Aoptome.2 microscope (Zeiss) as described above.

To stain *F. graminearum* wild-type mycelia, 2 µL (OD_600_ = 1) of conidia were germinated in 192 µL PGM at 28°C in the dark for 24h, then incubated with 6 µL of peptides for 3, 6, 12, and 24h and finally incubated with 200 µL NBT solution for 2h. After washing with PGM, mycelia were visualized as described above.

### Statistical analysis

An independent-samples *t*-test and an ANOVA were used to examine the effect of peptides on pollen tubes and fungal cells (where data were normally distributed) and an independent-samples *t*-test was used for the effect of the peptides (where data were not normally distributed). The data are presented as the corrected mean ±SD (*P* < 0.001).

## Results

### Structure of maize ES family peptides

**Fig. 1.**
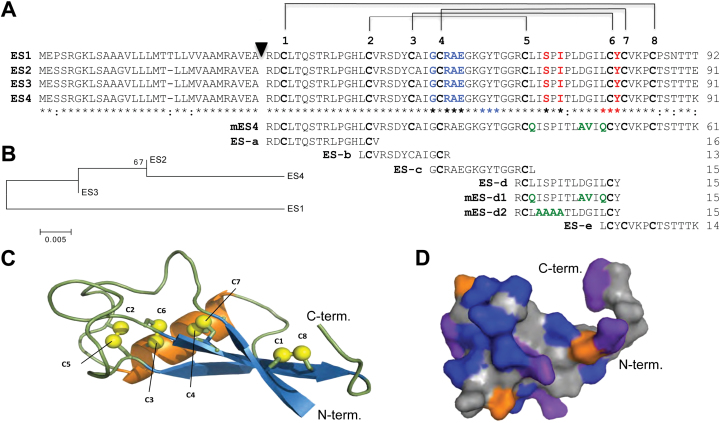
Sequences, phylogram, and 3D model of ES1–4 peptides and derived peptide fragments used in this study. (A) Amino acid sequences of maize ES1, ES2, ES3, and ES4 are highly similar and have in common a conserved defensin/DEFL motif of eight cysteines forming four intramolecular disulfide bridges. A black arrowhead marks predicted cleavage sites. Asterisks indicate identical amino acids of ES1–4 and double colons indicate mismatches. ES proteins were divided into five highly conserved peptide fragments (ES-a to ES-e including mutated fragments named mES) used for functional studies and mapping of interaction domains. Amino acids of ES-c and ES-d affecting maize pollen tube burst (see below) are highlighted in blue and red, respectively. Amino acids of ES-c and ES-d affecting growth of fungi are indicated by blue and red asterisks, respectively. (B) Protein sequences of ES1–4 were aligned by means of MEGA6.06 (Tamura *et al.*, 2013) and used to compute a maximum likelihood tree. The tree indicates the close relationship of ES2, ES3, and ES4 and the isolation of ES1. (C) Knottin structure of ES1–4 containing an antiparallel βαββ sheet and four disulfide bridges. (D) 3D structure of ES4 showing properties of individual surface residues. Colour code: grey, polar; blue, hydrophobic; orange, acidic; and purple, basic. The image was generated using PyMOL.

The sequences of ES1–4 peptides are highly similar to each other (Cordts *et al.*, 2001). ES4 shows a sequence identity of about 97% with ES2 and ES3, and of about 91% with ES1 (Fig. 1A, B). Three C-terminal mismatches distinguish mature peptides of ES1 and ES4 and another mismatch occurs in the hypervariable region ES-d (see below). Thus, predicted mature ES1 and ES4 peptides consisting of 61 amino acids were selected for functional studies. Mature ES family peptides were first divided into five highly conserved fragments named ES-a, ES-b, ES-c, ES-d, and ES-e (13–16 amino acids in length; Fig. 1A, B). These peptides and variants of ES-d were used for interaction and mapping studies. Moreover, considering that *ES1-4* genes encode peptides with structural homology to defensins and DEFLs, PyMOL (version 1.7.4; The PyMOL Molecular Graphics System, Version 1.7.4 Schrödinger, LLC) was used to generate homology models based on known 3D structures deduced from the protein data bank (Bernstein *et al.*, 1977). The 3D structure is characterized by a well-defined 3-stranded anti-parallel beta-sheet and a short alpha helix. Four disulfide bridges are located in the hydrophobic core between helix and sheet, forming a cysteine-stabilized alpha-helical motif (Fig. 1C). This structure is homologous to plant defensins and DEFLs, and analogous to scorpion toxins and insect defensins. However, an extended loop occurs in the ES-d peptide between Cys_5_ and Cys_6_. Therefore, the two variants mature ES-d1 (mES-d1) and mES-d2, as well as an altered mES4 peptide, were generated (Fig. 1A). For ES4, the electrostatic surface was calculated by means of PyMOL in order to visualize the distribution of charges and of hydrophobic and polar surfaces in an amino-acid-specific manner in 3D (Fig. 1D). The model shows that ES1–4 are hydrophobic peptides, contain a flexible C-terminus, and the domain containing ES-d is exposed from the surface of the molecule.

### Mapping of ES domains involved in pollen tube burst in maize

**Fig. 2.**
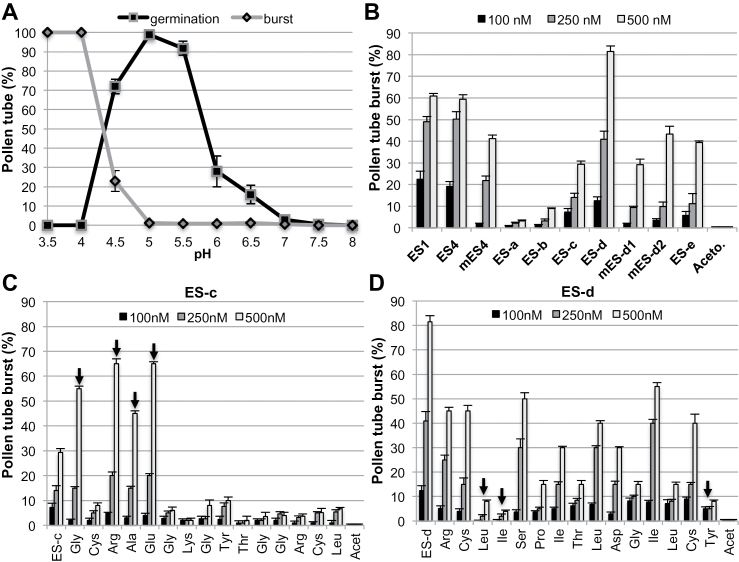
ES-d induces maize pollen tube burst. (A) Maize pollen grains were germinated in PGM at various pHs. Percentages of maize pollen tube germination and burst were measured after 1h at pH indicated. At pH 5 there was 100% germination with no burst. Error bars represent the standard error of 10 independent experiments. (B) Percentage of maize pollen tube burst was measured 5min after application of the indicated concentrations of peptides in PGM (pH 5). Acetonitrile was used as a negative control. (C) Percentage of maize pollen tube burst in PGM was measured 5min after application of ES-c and 15 mutated versions at the indicated concentrations. In each mutant version, the indicated amino acid was modified to Ala. Ala_4_ was modified to Val. Amino acids showing a strong increase in pollen tube burst are indicated by arrows. (D) Same experiment as shown in (C) but using ES-d and its variants. Amino acids reducing pollen tube burst below 10% are indicated by arrows. Error bars represent the standard error of eight independent experiments.

Previous studies have shown that the female gametophyte of maize likely secretes large amounts of ES1–4 required to induce pollen tube burst prior to fertilization (Cordts *et al.*, 2001; Amien *et al.*, 2010). ES1, ES4, mES4 (each 61 amino acid long); smaller peptide fragments ES-a, ES-b, ES-c, ES-d, and ES-e; and mutated versions mES-d1 and mES-d2 (13–16 amino acid long) were applied to growing pollen tubes in order to study their effects on pollen tube growth behaviour. First, the pH optimum of maize PGM was determined. Fig. 2A shows the germination and growth of maize pollen tubes for 1h at different pH values (pH 3.5 to pH 8). The maize pollen tubes germinated well, with between 92% and 99% germinating with no burst between pH 5 and 5.5. As a result, PGM at pH 5 was used for all further maize pollen tube assays. Different concentrations (100nM, 250nM, and 500nM) of peptides were applied to germinated maize pollen and showed that ES1 and ES4 induces maize pollen tube burst of 60.8±3.3% at 500nM (*P* < 0.001). Furthermore, a small peptide of 15 amino acids (ES-d) was even more active than full-length ES peptides and showed induction of pollen tube burst of 82.5±5.2% at 500nM. Peptides covering other ES domains showed no burst at all or a maximum burst efficiency of 39.3±2.4%. Mutants of ES-d (mES-d1 and mES-d2) and mutants of ES4 (mES4) showed significant reduction of pollen tube burst to about 29.2±7.0 to 41.2±4.4% (*P* < 0.001) (Fig. 2B). Moreover, after application of ES-d, maize pollen tubes started to burst within a few seconds, which is significantly faster than the response to mature peptides and other small peptides, in which the tubes started to burst after 1–2min. A series of concentrations was applied to obtain the optimal concentrations for the burst assay. Additionally, the time until burst was correlated with the concentration of the applied peptides. As a result, quantification of pollen tube burst was measured in the following experiments after 5min.

To identify amino acid residues involved in maize pollen tube burst and target interaction, ES-d (15 amino acids) and 15 mutated versions of ES-d were applied to germinated maize pollen at the above described concentrations. In each of these mutants, one amino acid was replaced by alanine (Ala). Analogously, ES-c (15 amino acids), which showed 29.3±4.2% pollen tube burst, was applied as well as 15 mutants in which one amino acid was replaced by Ala, while Ala in ES-c was replaced with valine (Val). The results showed that a mutation of Leu_3_, Ile_4_, or Tyr_15_ in ES-d suppressed maize pollen tube burst to <8±1.5% (*P* < 0.001) at a concentration of 500nM, suggesting that these amino acids are essential for target interaction. In contrast, a mutation of Gly_1_, Arg_3_, Ala_4_, or Glu_5_ in ES-c strongly increased induction of maize pollen tube burst to ~45±3.0 to 65±5.7% (Fig. 2C, D), indicating that ES peptides can be further modified to increase their activity/stability.

### ES-d binds at the surface of maize pollen tubes

**Fig. 3.**
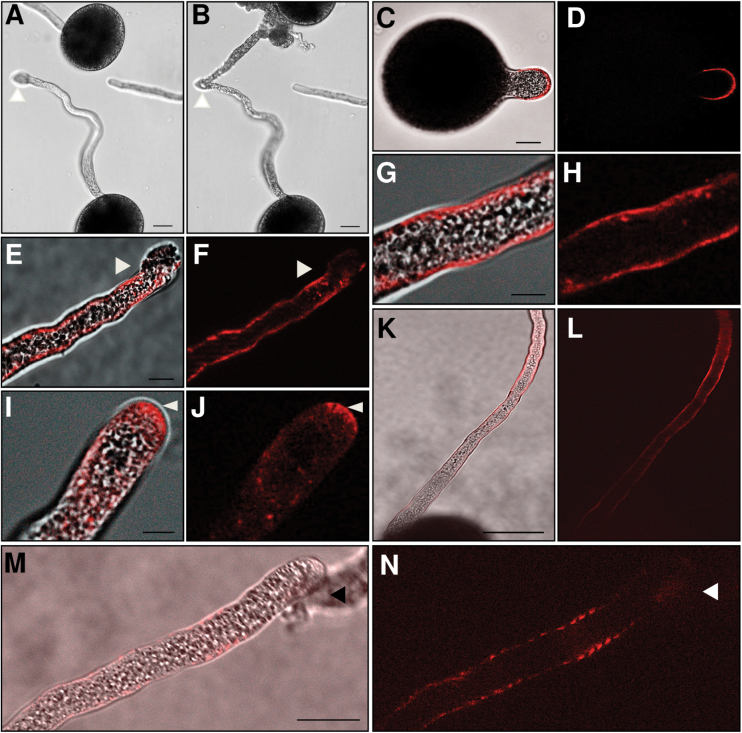
ES-d interacts with the surface of maize pollen tubes. (A, B) Maize pollen grains were germinated for 15min in PGM before addition of ES-d at 500nM. (A) A few seconds after ES-d application, pollen tube tips start swelling and immediately burst explosively at the apex (B). Arrowheads indicate swelling and burst. (C-J) Maize pollen grains were germinated for 15min in PGM before addition of TAMRA-ES-d at 50nM. Two minutes after TAMRA-ES-d application, labelled ES-d appears at the apex of germinating pollen tube (C, D) and throughout the whole pollen tube surface. Tubes burst explosively at the apex as indicated by arrowheads (E-F). Example showing TAMRA-ES-d bound along the pollen tube surface (G, H). Accumulation of TAMRA-ES-d (arrowheads) occurred at pollen tube tip preceding burst (I, J). (K, L) Binding of Rhodamin-ES4 at the pollen tube surface, and (M, N) bursting at the tip. (C, E, G I, K, M) show merge of bright field and fluorescence micrographs, while (D, F, H, J, L, N) show fluorescence micrographs. Scale bars are 50 µm in (K, L), 20 µm in (A-F, M, N) and 5 µm in (G-J).

It is difficult to investigate the binding of ES peptides at maize pollen tubes because application of ES-d (similar to ES1 and ES4) causes swelling of tube tips and growth arrest prior to explosive burst at the tube apex within a few seconds (Fig. 3A, B). To visualize the interaction and binding sites of ES-d on maize pollen tubes and to prolong swelling time to 2min prior to burst, a low concentration of 50nM of ES-d labelled with the fluorescent dye TAMRA was applied to germinated pollen tubes. The tubes showed fluorescence on the entire surface, which increased at the tips (Fig. 3C-J), causing swelling prior to explosive burst at the apex (Fig. 3E, F). Fluorescence was not detected on pollen grains. The same binding behaviour was observed after application of ES4 labelled with the fluorescent dye rhodamine. Here, burst occurred at the same concentration after 5min, indicating that longer peptides need more time to interact with the pollen tube surface (Fig. 3K-N). The same binding assay was repeated using ES-a and ES-c at the same concentration labelled with the fluorescent dyes TAMRA (ES-a) or Dabcyl (ES-c). For both assays, fluorescence was not observed in pollen grains and tubes (Supplementary Fig. S1).

### ES-c and ES-d inhibit fungal germination and growth and interact differently with targets compared with pollen tubes

**Fig. 4.**
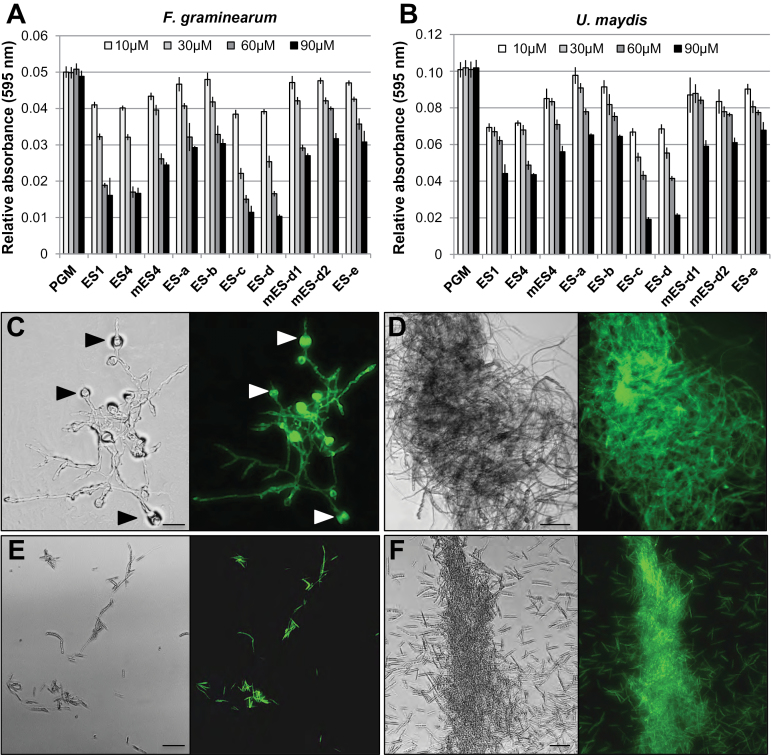
Germination inhibition of *F. graminearum* and *U. maydis* after application of ES-c or ES-d peptides. Germination of *F. graminearum* conidia (A) and *U. maydis* spores (B) was measured spectrophotometrically at 595nm with PGM for 24h after application of peptides (see Fig. 1) at indicated concentrations. ES-c and ES-d show strong and comparable inhibition of germination. Error bars represent the standard error of nine independent experiments. (C) Application of 30 µM ES-d for 24h at conidia of *F. graminearum*-3xGFP resulted in growth suppression and swelling of mycelia (arrowheads) compared with (D) the control lacking peptides. (E) Application of 60 µM ES-d at spores of *U. maydis*-3xGFP resulted in strong suppression of germination compared with (F) the untreated control. Micrographs (C-F) show bright field at the left side and fluorescence at the right side. Scale bars are 20 µm in (C) and 50 µm in (D-F).

Considering that maize ES1–4 are cysteine-rich peptide precursors belonging to the plant defensin/DEFL group, a fungal germination inhibitory assay was applied to study their antifungal activity and defence functions. Two maize fungal pathogens of different infection types were tested: the necrotrophic fungus *F. graminearum* and the biotrophic fungus *U. maydis* (CIMMYT, 2004; Dean *et al.*, 2012). Thus, *Fusarium* conidia and *Ustilago* spores were inoculated *in vitro* with increasing concentrations of ES peptides. To quantify fungal biomass production and growth, *F. graminearum*-3xGFP and *U. maydis*-3xGFP strains that constitutively express triple versions of the gene for GFP were used. *Fusarium* conidia and *Ustilago* spores were inoculated in PGM, which was supplemented with varying concentrations of peptides. Peptide concentrations of 0.1–0.5 μM used for pollen tube burst assays did not show effects on fungal growth behaviour. However, strongly increased concentrations of 10–90 μM demonstrated a dose-dependent inhibition of germination after 24h. Remarkably, 90 μM of ES1 inhibited 66.7±1.1%, and ES4 inhibited 67.8±1.5% of the germination of *Fusarium* conidia, while ES-c inhibited 77.1±1.1% and ES-d inhibited 79.3±1.6% of conidia (*P* < 0.001). In *Ustilago* spores, 90 μM of ES1 inhibited 55.9±0.9% of germination and ES4 inhibited 56.4±0.9%, while ES-c inhibited 80.9±1.0% and ES-d inhibited 78.7±1.0% (*P* < 0.001). A significant inhibition was not observed for other small peptides and their variants (Fig. 4A, B). Conidia and spores showed a severely malformed appearance at a concentration of 90 µM of ES-c or ES-d. Approximately 80% of conidia and spores did not germinate at all, while the rest stopped growing after forming a short germination mycelium. Complete suppression of conidia and spore germination was observed at 120 μM. A peptide concentration of 10 µM resulted only in minor fungal growth differences. In contrast, at concentrations of 30–60 µM, a reduction in germination and fungal growth occurred, including severe swelling and malformed appearance (Fig. 4C-F). Moreover, *Fusarium* mycelium developed from germinated conidia failed to form hyphal colonies. Instead, they showed swelling and ballooning at mycelia tips, in comparison to untreated wild-type conidia, which showed development of normal long and thin filaments (Fig. 4C, D). Swelling and ballooning was not observed after peptides were applied to *Ustilago* spores, but germination of mycelium was strongly inhibited (Fig. 4E, F).

To determine the most important amino acid residues involved in inhibiting fungal germination, ES-c and ES-d as well as 15 mutant versions of ES-c or ES-d (see above) were applied to *Fusarium* conidia and *Ustilago* spores as described. Mutations of Gly_8_, Tyr_9_, or Thr_10_ in ES-c (Supplementary Fig. S2A) and mutations of Lys_13_, Cys_14_, or Tyr_15_ in ES-d (Supplementary Fig. S2B) significantly increased fungal germination to 66.7±3.3% to 78±2.2% in *Fusarium*. A similar result was obtained in *Ustilago.* Mutations of the same amino acids increased germination of spores to 67.2±1.3% to 72.6±1.4% (*P* < 0.001) compared with the controls at a concentration of 90 µM (ES-c and ES-d, respectively; Supplementary Fig. S3A, B). Germination above 60% was considered as a threshold for both fungi. ES-c and ES-d showed fungal germination of 19.1±3.8% to 20.6±2.1%, respectively, at the same conditions in both fungi (Supplementary Figs. S2 and S3). In conclusion, ES peptides showed similar effects on fungal cells compared with maize pollen tubes. However, significantly higher concentrations (about 100×) had to be applied to observe comparable effects. Moreover, in contrast to pollen tubes, both ES-c and ES-d showed activity on fungal cells.

### ES-c and ES-d bind differently to fungal cells and induce ROS production

**Fig. 5.**
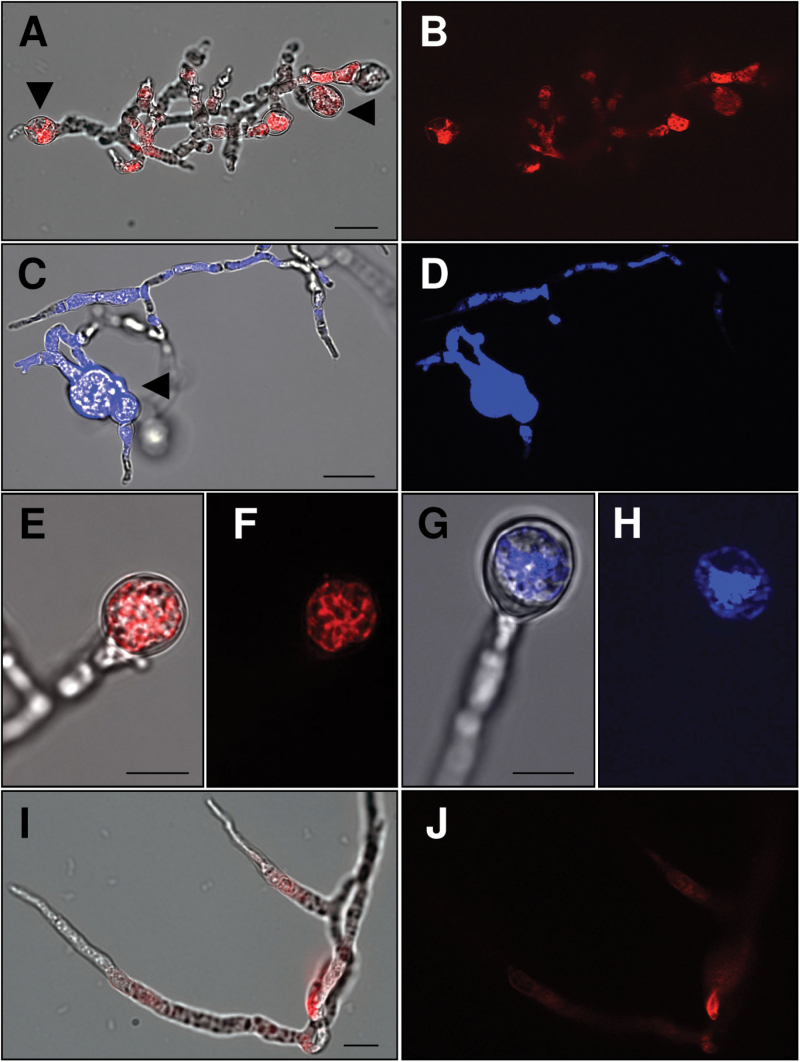
ES-c and ES-d peptides bind *F. graminearum* cells and induce their swelling. Application of 30 µM TAMRA-ES-d (A, B) or Dabcyl-ES-c (C, D) for 24h at conidia of *F. graminearum* showed their accumulation at growing mycelia, suppression of conidia germination, and growth as well as swelling and ballooning of cells (arrowheads). (E-H) Examples showing swelling of mycelium tips and accumulation of labelled peptides after application of 60 µM TAMRA-ES-d (E, F) or Dabcyl-ES-c (G, H). (I-J) Application of 30 µM TAMRA-ES-a for 24h at *F. graminearum* conidia resulted in normal conidia germination and mycelial growth. Only traces of labelled peptide are visible. Micrographs (A, C, E, G, I) show merge of bright field and fluorescence, while micrographs (B, D, F, H, J) show fluorescence. Scales bars are 20 µm in (A-D, I-J) and 10 µm in (E-H).

**Fig. 6.**
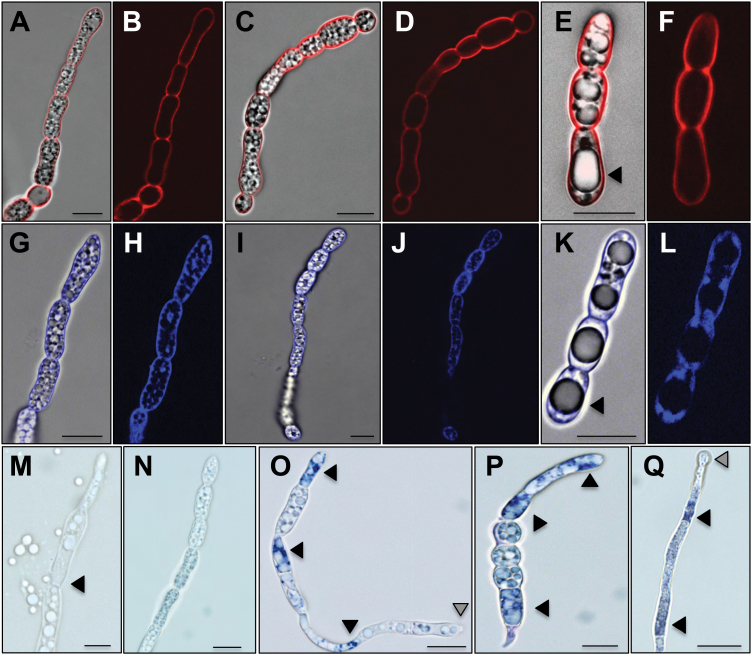
Localization of ES-c and ES-d at the surface of mycelia and conidia of *F. graminearum*, ROS detection, and mycelium burst. (A, B) Conidia were germinated in PGM for 24h before addition of TAMRA-ES-d at 60 µM. Three hours after application, labelled peptide appeared at the mycelium surface. (C, D) Some conidia showed swelling of cells. (E, F) Three hours after application of 90 µM TAMRA-ES-d, conidia showed extreme swelling, enlargement of cells,and formation of big vacuoles (arrowheads). (G, H) Conidia were germinated in PGM for 24h before addition of Dabcyl-ES-c at 60 µM. Three hours after application, labelled ES-c appeared at the mycelium surface and intercellular surfaces. (I, J) Some conidia showed swelling of cells. (K, L) Three hours after application of 90 µM Dabcyl-ES-c, conidia showed extreme swelling, enlargement of cells, and formation of big vacuoles (arrowheads). ES-c binds at the conidia surface and intercellular surfaces and appears inside cells. (M) Six hours after application of 90 µM ES-d, burst of swelled mycelium occurs (arrowhead). (N) ROS was not detectable by NBT in conidia treated for 24h with 60 µM ES-a. (O, P) ROS was detected in swelling cells of conidia treated for 24h with 60 µM ES-d (arrowheads) (O) or ES-c (P). Excretion of vacuoles and ROS at germinated conidia tips (grey arrowhead) occurred with ES-d (O). (Q) ROS was detected in swelling cells (arrowheads) and swelling tips (grey arrowhead) of mycelium treated with 60 µM ES-d for 6h. Micrographs (A, C, E, G, I, K) show merge of bright field and fluorescence, while micrographs (B, D, F, H, J, L) show fluorescence. (M-Q) Bright field micrographs. Scales bars are 10 µm in (A-Q).

To detect and visualize the effect and interaction of ES-c and ES-d on germinated *Fusarium* conidia and developed mycelium, conidia of *F. graminearum* were inoculated in PGM following the addition of final concentrations of 30–90 μM of labelled peptides Dabcyl-ES-c, TAMRA-ES-d, and Rhodamine-ES4, as well as TAMRA-ES-a as a control at the four different stages according to Woriedh *et al.* (2011): (i) fresh conidia (0 hours post induction, hpi), (ii) activated conidia (3 hpi), (iii) germinated conidia (6 hpi), and (iv) developed hyphae (24 hpi), respectively. The analysis of localization and binding of Dabcyl-ES-c, TAMRA-ES-d, and Rhodamine-ES4 on germinated conidia and developed mycelium showed a dose- and time-dependent inhibition, as mentioned above. Germinated conidia took up labelled peptides and accumulated them 12 and 24 hpi in swollen and ballooned compacted cells of developed mycelia. In general, *Fusarium* mycelia appeared compacted, shorter, and thicker, and contained swollen tips (Fig. 5A-H) compared with germinated conidia treated with TAMRA-ES-a, where a weak accumulation of fluorescence was observed (Fig. 5I, J). Here, developed mycelia showed normal hyphal growth of thin and long branched filaments. Observation at earlier time points at 3 and 6h was also conducted: TAMRA-ES-d was localized and bound only at the surface of activated conidia, germinated conidia, and developed mycelia (24 hpi), while Dabcyl-ES-c bound at the surface and accumulated inside activated conidia, germinated conidia, and developed mycelia (Fig. 6A-D and G-J). Furthermore, increasing the concentration to 90 µM resulted in swelling and enlargement of fresh conidia and formation of big vacuoles, which finally fused to a single large vacuole within the cells, forcing conidia to stop germination. Although TAMRA-ES-d bound only at surfaces of vacuolated conidia, Dabcyl-ES-c was detected both at cell surfaces and appeared to be inside the cell in vacuolated conidia (Fig. 6E, F, K, L). Increasing the concentration of ES-c or ES-d to 90 µM in developed mycelia (24 hpi) caused, after 6h, swelling of cells, formation of vacuoles, and subsequent breaking/bursting of cell walls and release of vacuoles (Fig. 6M). In contrast, fresh conidia, germinated conidia, and developed conidia showed normal growth behaviour and lack of fluorescence 3h and 6h after application of the control peptide TAMRA-ES-a at concentrations of 30–90 µM (Supplementary Fig. S4). Application of Rhodamine-ES4 showed inhibition behaviour similar to ES-c and ES-d. At the same time, fluorescence was observed at the cell surface and accumulated inside conidia and mycelia. Furthermore, conidia and mycelia treated with ES-a, ES-c, ES-d, or ES4 were also stained with NBT to detect the production of ROS. NBT reacts with superoxide (O_2_^-^) anions to form a dark blue insoluble formazan compound. Conidia and mycelia treated with ES-c, ES-d, or ES4 strongly produced ROS. In addition, swollen and vacuolated conidia and mycelia were formed. Staining of conidia and mycelia treated with ES-a showed weak to no traces of ROS (Fig. 6N-Q).

**Fig. 7.**
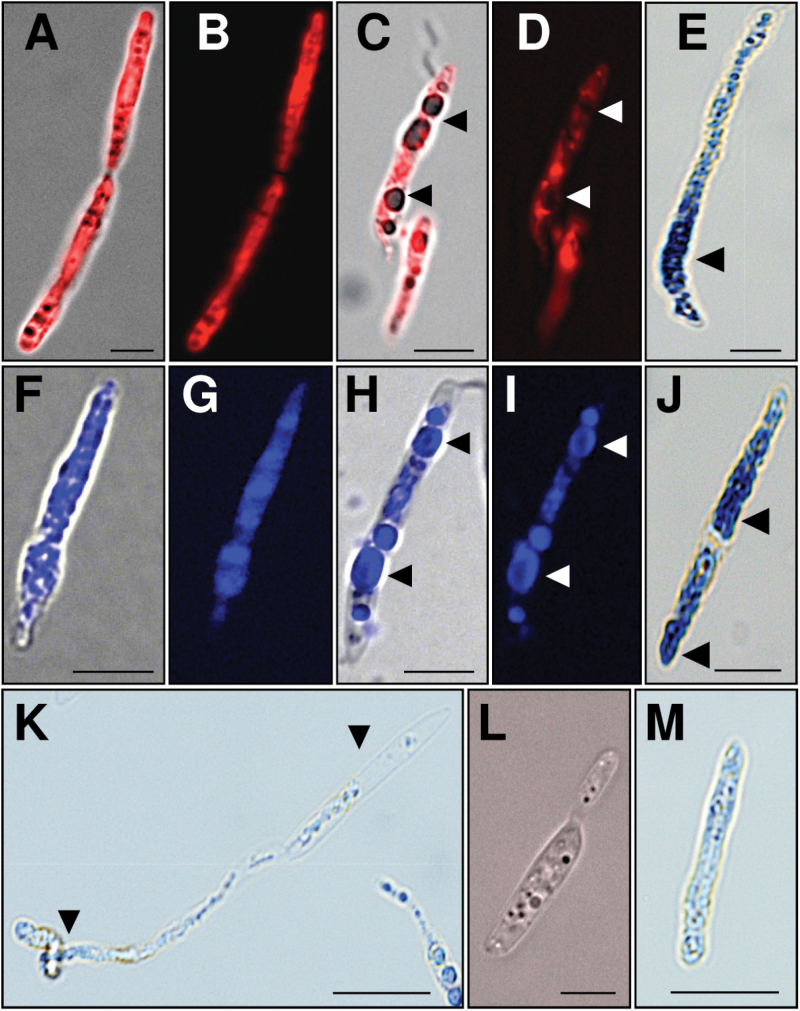
Accumulation of ES-c and ES-d at spores of *U. maydis*, ROS detection, and spore burst. (A, B) Spores were germinated in PGM for 24h before addition of TAMRA-ES-d at 30 µM. Three hours after application, ES-d appeared in whole spores. (C, D) Three hours after application of 90 µM TAMRA-ES-d, spores showed swelling and enlargement of cells and formation of big vacuoles (arrowheads). (E) ROS were detected by NBT in spores treated for 24h with 60 µM of ES-d (arrowhead). (F, G) Spores were germinated in PGM for 24h before addition of Dabcyl-ES-c at 30 µM. Three hours after application, ES-c appeared in whole spores. (H, I) Three hours after application of 90 µM Dabcyl-ES-c, spores showed swelling, enlargement of cells, and formation of big vacuoles (arrowheads). ES-c accumulated inside vacuoles and fluorescence was not detected at the surface of spores. (J) ROS was detected by NBT in spores treated for 24h with 60 µM ES-c (arrowhead). (K) Six hours after application of 90 µM ES-d there was burst of swelling spores and excretion of vacuoles and cell components (arrowheads). (L) Spores were germinated in PGM for 24h before addition of TAMRA-ES-a at 30 µM. Three hours after application, fluorescence was not detectable and spores showed normal growth behaviour. (M) Traces of ROS were detected in spores treated for 24h with 60 µM ES-a. Micrographs (A, C, F, H, L) show merge of bright field and fluorescence, while micrographs (B, D, G, I) show fluorescence. (K, M) Bright field micrographs. Scales bars are 5 µm in (A-J, L) and 10 µm in (K, M).

All of the above described assays for localization and binding were also applied to *U. maydis* in order to quantify the effect of ES-c and ES-d on *Ustilago* spores. Similar to *Fusarium*, binding and activity of Dabcyl-ES-c, TAMRA-ES-d, and Rhodamine-ES4 on germinated *Ustilago* spores showed a dose- and time-dependent inhibition. *Ustilago* spores treated with 30 or 60 µM Dabcyl-ES-c and TAMRA-ES-d showed an accumulation of fluorescence. Spores became thicker and bigger than untreated wild-type spores and mycelia germination was inhibited or prevented completely (Fig. 7A, B, F, G). At a concentration of 90 µM, treated spores were malformed within 3h. Spores appeared swollen and enlarged and formed many vacuoles, which later assembled into few large vacuoles. TAMRA-ES-d was detected in whole spores while Dabcyl-ES-c was detected mainly in vacuoles (Fig. 7C, D, H, I). After 6h, swollen and vacuolated cells started to burst, releasing vacuoles and cellular components (Fig. 7K). In contrast, germinated spores, which were treated with the control TAMRA-ES-a, showed normal spore growth behaviour and fluorescence could not be detected (Fig. 7L). Application of Rhodamine-ES4 resulted in the same inhibition behaviour as ES-c and ES-d. Fluorescence was observed in whole spores and in vacuoles. Furthermore, ROS production was also detected by NBT in spores treated with ES-c, ES-d, and Rhodamine-ES-4. Germinated *Ustilago* spores produced significant ROS amounts after application of 30 and 60 µM of the above-mentioned peptides (Fig. 7E, J). Traces of ROS were detected when ES-a was applied (Fig 7M).

### ES-d loop domain is maize-specific

**Fig. 8.**
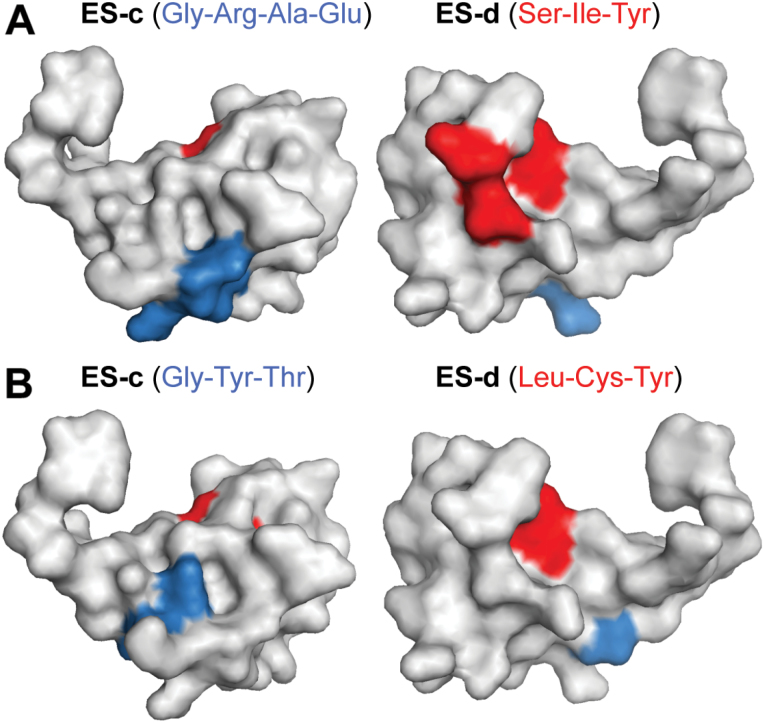
3D models showing the most important amino acid residues in mediating maize pollen tube burst and fungal growth inhibition. (A) 3D structure of ES4 (front and back view) showing amino acids of ES-c in blue and ES-d in red, whose exchanges increased (blue) and decreased (red) maize pollen tube burst. (B) 3D structure of ES4 showing amino acids of ES-c in blue and ES-d in red, whose exchanges suppressed inhibition of conidia and spore germination, respectively. 3D structures were generated with PyMOL.

A protein sequence comparison of ES1–4 and entries in the RefSeq protein database showed that most regions of the ES family (including the peptide signal cleavage site) are well conserved in plant defensins and DEFLs. However, while ES-e (C-terminal region) is less conserved, the ES-d region has no similarity with other defensins/DEFLs and is specific for maize. Furthermore, amino acid residues of ES-c and ES-d targeting fungal growth inhibition are more conserved within plant defensins/DEFLs. In contrast, ES-c and ES-d residues affecting maize pollen tube burst are highly conserved within the ES family, but less in other plant defensins/DEFLs (Supplementary Fig. S5 and Table S1). In order to indicate the localization of important residues, Fig. 8 shows the 3D model of ES4 in two different orientations. Gly_1_, Arg_3_, Ala_4_, and Glu_5_ in ES-c, and Leu_3_, Ile_4_, or Tyr_15_ in ES-d affect pollen tube burst, while Gly_8_, Tyr_9_, and Thr_10_ in ES-c, and Lys_13_, Cys_14_, and Tyr_15_ in ES-d affect germination and inhibition in fungi. As shown, there is little overlap, indicating that ES peptides interact differentially or with different targets at the surface/cell wall of pollen tubes and fungal cells.

## Discussion

A previous study (Amien *et al.*, 2010) showed that extracellular cysteine-rich peptide precursors such as a Bowman–Birk trypsin inhibitor from soybean (*Glycine max*), pollen tube attractant LURE2 of wishbone flower (*Torenia fournieri*), or anti-fungal protein AFP2 from radish (*Raphanus sativus*) did not affect maize pollen tube growth behaviour, and thus pointed towards a very specific activity of ES peptides. Moreover, application of ES4 caused a significant delay of pollen tube rupture in the close maize relative eastern gamagrass (*Tripsacum dactyloides*) but did not have any effect on tubes of unrelated maize species such as tobacco (*Nicotiana benthamiana* and *N. tabaccum*) or lily (*Hemerocallis fulva*). These observations further indicate that ES peptides are species-specific. In the present study, these findings were extended to show that other ES members (ES1–3) are capable of inducing maize pollen tube burst in the same manner. Additionally, an ES peptide fragment that is specific for maize, named ES-d, was found to bind at the pollen tube surface, accumulate at tube tips, and cause swelling prior to burst. These effects were not observed for other less-specific ES peptide fragments. ES-d contains a putatively exposed loop region of the mature peptide as well as amino acids essential for the induction of pollen tube burst in maize. Thus, ES-d represents the most likely active region of the peptide that physically interacts with receptor(s) at the pollen tube surface. The specificity of this region, which is also different from corresponding regions of homologous peptides of other grasses, suggests that it may have played an important role during speciation and the formation of the genus *Zea*. Future experiments using pollen tubes from other grasses and the identification of orthologous proteins in incompatible maize inbred lines and closely related species such as *T. dactyloides* will help to prove this hypothesis and thus may become important in overcoming hybridization barriers among grass species.

At significantly higher concentrations (10 µM and above), ES peptide fragments were found to possess broad antifungal activity. However, inhibition of cereal rust fungi, for example, is usually achieved by defensins NaD1 and NaD2 from flowers of tobacco (*N. alata*) at a concentration of 1–2 µM (Dracatos *et al.*, 2014). Peach (*Prunus persica*) defensin DFN1 also displayed antifungal activity at 1 µM through specific interactions with membrane lipids of *Botrytis cinerea*, *Penicillium expansum*, and *Monilinia laxa* (Nannia *et al.*, 2012). These findings indicate that ES peptides have lost most of their antifungal activity. Antimicrobial activity of ES4 has been studied previously, but direct antibacterial and antifungal activities against *Pseudomonas syringae* pv. *tomato* DC3000 (*Pto* DC3000) and *Hyaloperonospora arabidopsidis* (previously known as *Peronospora parasitica*), respectively, were not observed in *in vitro* experiments (Amien *et al.*, 2010). However, when continuously expressed at high amounts under regulation of the *35S* promoter, *ES4*-overexpressing seedlings infected with *H. arabidopsidis* recovered faster than controls. Additionally, fungal hyphae were no longer macroscopically visible a few weeks after infection. The mild effect on *H. arabidopsidis*, an obligate parasite and causal agent of the downy mildew of the plant model organism *Arabidopsis thaliana* (Schlaich and Slusarenko, 2009; Fry and Grünwald, 2010), might, however, also be due to the fact that this pathogen is unspecific for maize and other grasses. In contrast, ES1–4 showed a high and significant activity of two fungal pathogens of maize in the present study. Both fungi cause severe diseases during flowering time in maize through infection of reproductive tissues (Banuett, 1995; Klosterman *et al.*, 2007; Shea Miller *et al.*, 2007; Dean *et al.*, 2012). Interestingly, both ES-c and ES-d peptides showed antifungal responses, but they bound differently to *Fusarium* and *Ustilago* and even differently among fungus cell types. These experiments indicate that ES peptides likely interact with multiple receptors/targets in fungi.

In this study, applications of ES1–4 resulted in swelling and rupture of both pollen tube and fungal cells. Swelling and vacuole formation (in fungal cells) culminating in rapid cell rupture points towards water uptake. This may be caused by altered solute transport across the plasma membrane and/or increased membrane permeability. Killing of cells by antimicrobial peptides has been proposed to occur in two main ways: (i) through disruption of the plasma membrane leading to leakage of cytoplasmic contents or (ii) through interaction with intracellular targets (Brogden, 2005). The results from this study suggest that cell death occurred through both ways because peptides localized first at the cell surface then later inside cells. The plant defensin NaD1 from tobacco (*N. alata*) flowers was previously shown to display a potent antifungal activity against a variety of agronomically important filamentous fungi including *F. oxysporum* f. sp. *vasinfectum* (Fov). NaD1 entering the cytoplasm of hyphae results in granulation of the cytoplasm and cell death through ROS production (Van Der Weerden *et al.*, 2008) similar to the activity of the studied ES peptides. NaD1 and the antifungal defensin AFP2 from seed of *Raphanus sativus* were also shown to possess activity against the major human pathogen *Candida albicans.* The mechanism involves interaction between NaD1 or AFP2 and the fungal cell surface followed by membrane permeabilization, entry into the cytoplasm, production of ROS, and cell death induced by oxidative damage (Aerts *et al.*, 2007; Hayes *et al.*, 2013). It is still unknown whether permeabilization by ES-c or ES-d causes cell death by inducing leakage of cytoplasmic contents, by ion fluxes, or by facilitating entry of peptides into cells to access intracellular targets.

Plant defensins possess a variety of biological activities beside inhibition of ion channel function and have been reported, for example, to inhibit protein synthesis and enzyme activity (Vriens *et al.*, 2014). Using the microelectrode impalement technique, Amien *et al.* (2010) showed that ES4 application leads to H^+^ and/or K^+^ flux and that ES4 target(s) likely represent ion-channel(s). Detailed studies expressing the maize potassium channel *KZM1* in *Xenopus* oocytes showed that ES4 application leads to opening of the channel, which is almost closed at physiological conditions. The current model suggests that opening of KZM1 leads to flux of the strong osmoticum K^+^, which may cause membrane depolarization, but which may also influence the conductivity of a battery of voltage-dependent transporters located at the pollen tube plasma membrane and, ultimately, culminate in osmotic burst (Dresselhaus and Franklin-Tong, 2013). ES targets localize almost uniformly at the surface (likely plasma membrane) of the pollen tube, displaying some accumulation at the very tip of the pollen tube. This pattern further indicates that KZM1 may not represent the only interactor as its accumulation in the tip was not observed in pollen tubes transiently transformed with KZM1.

ES peptides also strongly inhibit the growth of *F. graminearum* and *U. maydis in vitro*, indicating that ES peptides may be able to regulate channels of the KZM1 type in fungal cells, but may also interact with other yet unknown channels. The maize defensin subclass of γ-zeathionins, for example, was shown to block Na^+^ channels in animal cells (Kushmerick *et al.*, 1998) and defensin MsDef1 from alfalfa (*Medicago sativa*) seeds was reported to block the mammalian L-type Ca^2+^ channel. Notably, antifungal activity of MsDef1 is markedly reduced in the presence of Ca^2+^ (Spelbrink *et al.*, 2004). Treatment of hyphae of *Neurospora crassa* with antifungal plant defensins, for example with Rs-AFP2, Dm-AMP1, and α-hordothionin isolated from radish, dahlia (*Dahlia merckii*), or barley (*Hordeum vulgare*) seeds induced a rapid K^+^ efflux, Ca^2+^ uptake, and alkalinization of the incubation medium, suggesting that plant defensins act on multiple channels and/or receptors (Thevissen *et al.*, 1996; Vriens *et al.*, 2014). K^+^ channels were recently also found to be inhibited by animal toxin-like human β-defensin 2, which interacts with the extracellular channel pore region (Yang *et al.*, 2015). The K^+^ channel-blocking reagent plectasin, a defensin blocker from the fungus *Pseudoplectania nigrella*, could block Kv1.3 channel currents in a dose-dependent manner. Besides Kv1.3, plectasin could also inhibit other channels like Kv1.1, Kv1.2, IKCa, SKCa3, hERG, and KCNQ with less activity (Xiang *et al.*, 2015). Scorpion defensins, analogues of ES1–4, were also reported to affect functions of multiple ion channels for Na^+^, K^+^, and Ca^2+^ (Quintero-Hernandez *et al.*, 2013). Together, these findings not only highlight the function of defensins as ion channel targets, but also imply that defensins from different organisms functionally evolved different ion channels as targets. How ES1–4 peptides interact with fungal hyphae or with maize pollen tubes to generate plasma membrane ion fluxes remains unclear and needs further investigation.

The picture may become even more complex, as defensins/DEFLs also interact with receptor-like kinases. In *A. thaliana*, for example, TAPETUM DETERMINANT 1, EPIDERMAL PATTERNING FACTOR (EPF), and related EPF-Like, and S-LOCUS CYSTEINE RICH proteins were shown to interact with receptor-like kinases rather than ion channels (for review see Butenko and Aalen, 2012). To understand the complexity of ES activity and the specificity of its subdomains, a systematic, preferentially biochemical, approach is now necessary to elucidate all interaction partners of ES peptides both in pollen tubes and fungal cells.

## Supplementary data

Supplementary data are available at *JXB* online.

Fig. S1. ES-a and ES-c peptides do not bind to maize pollen tubes.

Fig. S2. Identification of amino acid residues in ES-c and ES-d peptides affecting germination of *Fusarium graminearum*.

Fig. S3. Identification of amino acid residues in ES-c and ES-d peptides affecting germination of *Ustilago maydis.*

Fig. S4. ES-a peptide does not bind to *F. graminearum.*

Fig. S5. ES family peptides lack homology to plant defensins/DEFLs in regions responsible for their pollen tube burst activity.

Table S1. List of aligned plant defensin/DEFL proteins homologous to ES1–4.

Supplementary Data
