# Supplementary material for: Maize EMBRYO SAC family peptides interact differentially with pollen tubes and fungal cells
Source: J Exp Bot. 2015 Jun 12;66(17):5205–16. doi: 10.1093/jxb/erv268 (PMC4526917; doi:10.1093/jxb/erv268)
Supplement: Supplementary Data [file supp_66_17_5205__index.html]

Maize EMBRYO SAC family peptides interact differentially with pollen tubes and fungal cells — Maize EMBRYO SAC family peptides interact differentially with pollen tubes and fungal cells — Supplementary Data 

# Maize EMBRYO SAC family peptides interact differentially with pollen tubes and fungal cells

## Supplementary Data

Data files

- Supplementary Data - Supplementary Data
- Supplementary Data - Supplementary Data
