# Supplementary material for: Maize EMBRYO SAC family peptides interact differentially with pollen tubes and fungal cells
Source: J Exp Bot. 2015 Jun 12;66(17):5205–16. doi: 10.1093/jxb/erv268 (PMC4526917; doi:10.1093/jxb/erv268)
Supplement: Supplementary Data [file supp_erv268_Supplementary_Figures_Woriedh_et_al_rev2.pdf]

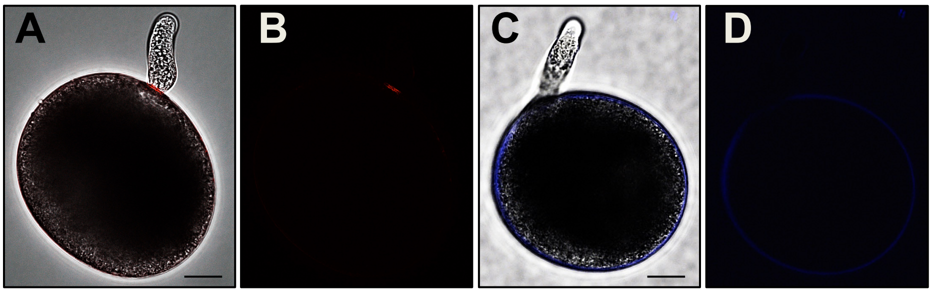

Figure S1. ES-a and ES-c peptides do not bind to maize pollen tubes. Pollen grains were germinated for 15 min in PGM before addition of TAMRA-ES-a (A-B) or Dabcyl-ES-c (C-D) at 50 nM in PGM (pH 5). Pollen tubes display normal growth behavior and lack of fluorescence. (A) and (C) show merge of bright field and fluorescence micrographs. (B) and (D) show fluorescence micrographs. Scale bars are 20 μm.

Fusarium graminearum

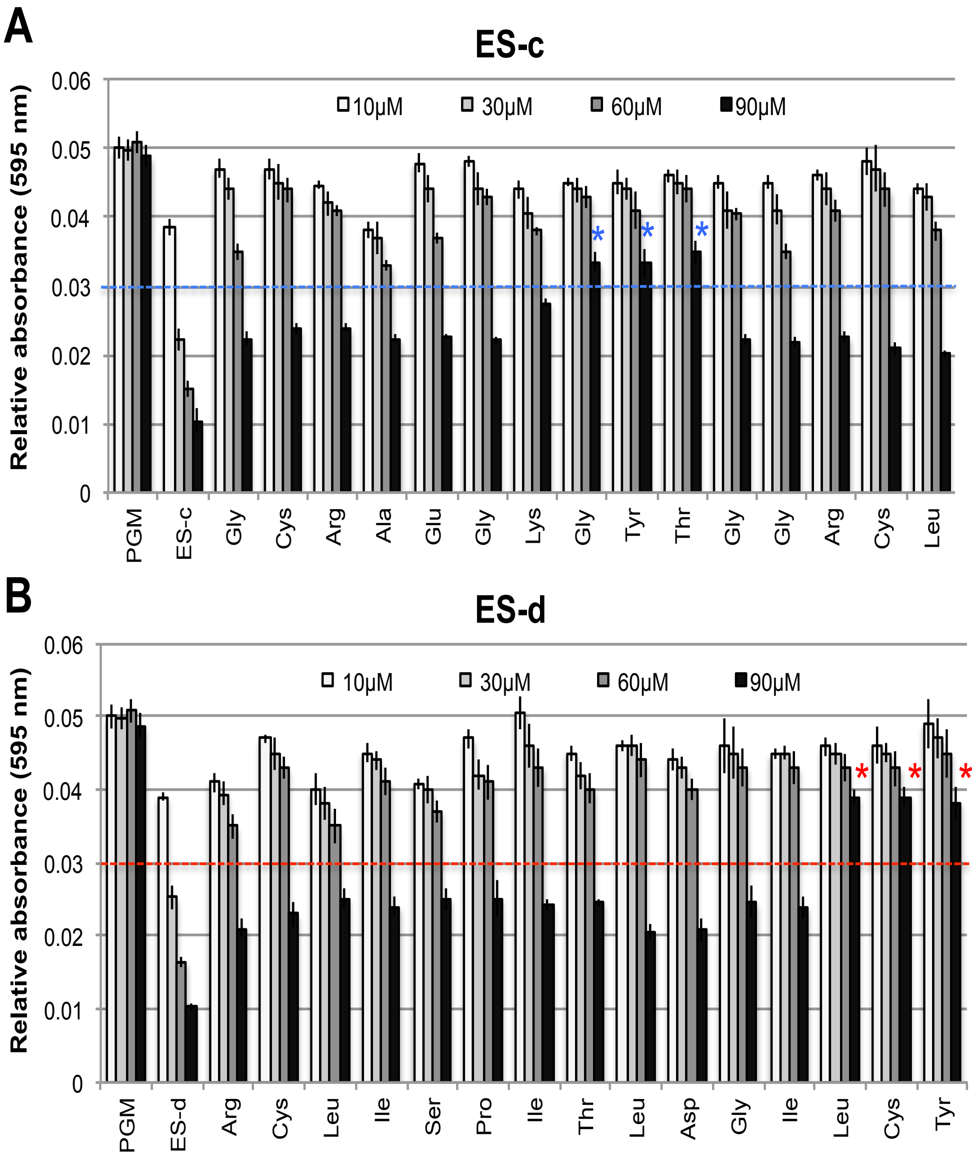

Figure S2. Identification of amino acid residues in ES-c and ES-d peptides affecting germination of *Fusarium graminearum*. Germination of *F. graminearum* conidia was measured spectrophotometrically at 595 nm in 96 plates with PGM 24 hours after application of 10 µM, 30 µM, 60 µM and 90 µM of ES-c and 15 mutated versions of ES-c in (A), or after application of ES-d and 15 mutated versions of ES-d in (B). Each one of the indicated amino acids was mutated to Ala. Ala_4 of ES-c was mutated to Val. Alterations of Gly, Tyr and Thr in ES-c (blue asterisks) as well as Leu, Cys and Tyr in ES-d (red asterisks) are above 60%, a threshold for conidia germination indicated by blue and red dashed lines. Thus these residues appear to play a major role in inhibition of *Fusarium* germination. Error bars represent the standard error of nine independent experiments.

Ustilago maydis

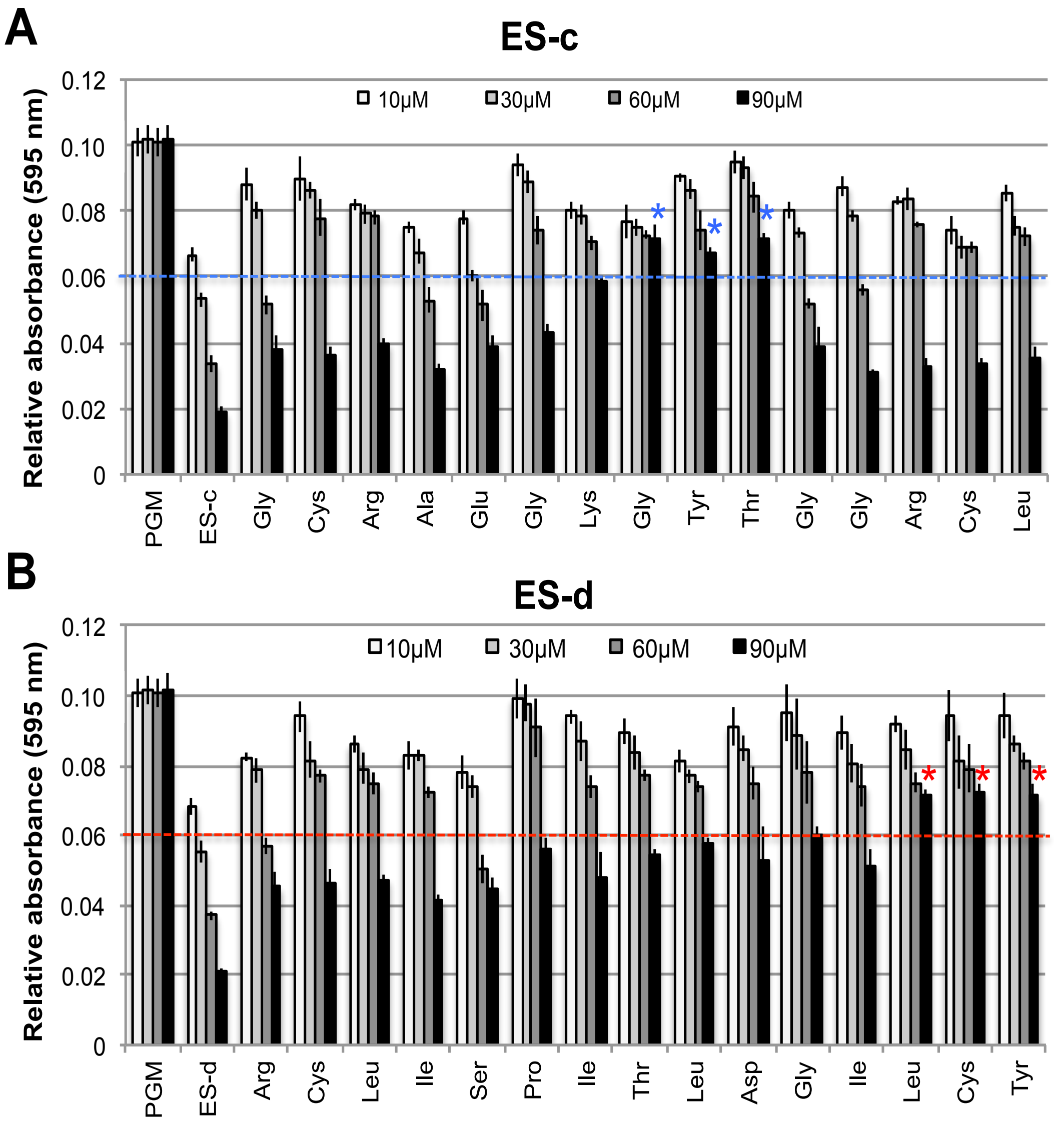

Figure S3. Identification of amino acid residues in ES-c and ES-d peptides affecting germination of *Ustilago maydis*. Germination of *U. maydis* spores was measured spectrophotometrically at 595 nm in 96 plates with PGM 24 hours after application of 10 µM, 30 µM, 60 µM and 90 µM of ES-c and 15 mutated versions of ES-c in (A), or after application of ES-d and 15 mutated versions of ES-d in (B). Each one of the indicated amino acids was only mutated to Ala. Ala_4 of ES-c was mutated to Val. Alterations of Gly, Tyr and Thr in ES-c (blue asterisks) as well as Leu, Cys and Tyr in ES-d (red asterisks) are above the threshold of 60%. This threshold is indicated with blue and red dashed lines. We suggest that these residues play a major role in inhibition of *Ustilago* germination. Error bars represent the standard error of nine independent experiments

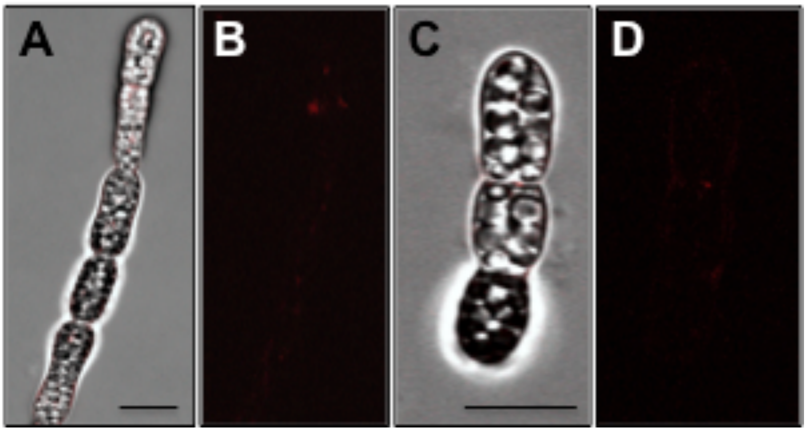

Figure S4. ES-a peptide does not bind to *Fusarium graminearum.* *Fusarium* conidia were germinated in PGM for 24 h before addition of TAMRA-ES-a at 60 µM. (A-B) 3 h after application, germinated conidia display normal mycelium growth behavior and lack of fluorescence. (C-D) Another conidia showing a normal phenotype and lack of fluorescence. (A) and (C) show merge of bright field and fluorescence micrographs. (B) and (D) show fluorescence micrographs. Scale bars are 10 µm.

"Maize ES family peptides interact differentially with pollen tubes and fungal cells"

M. Woriedh, R. Merkl & T. Dresselhaus

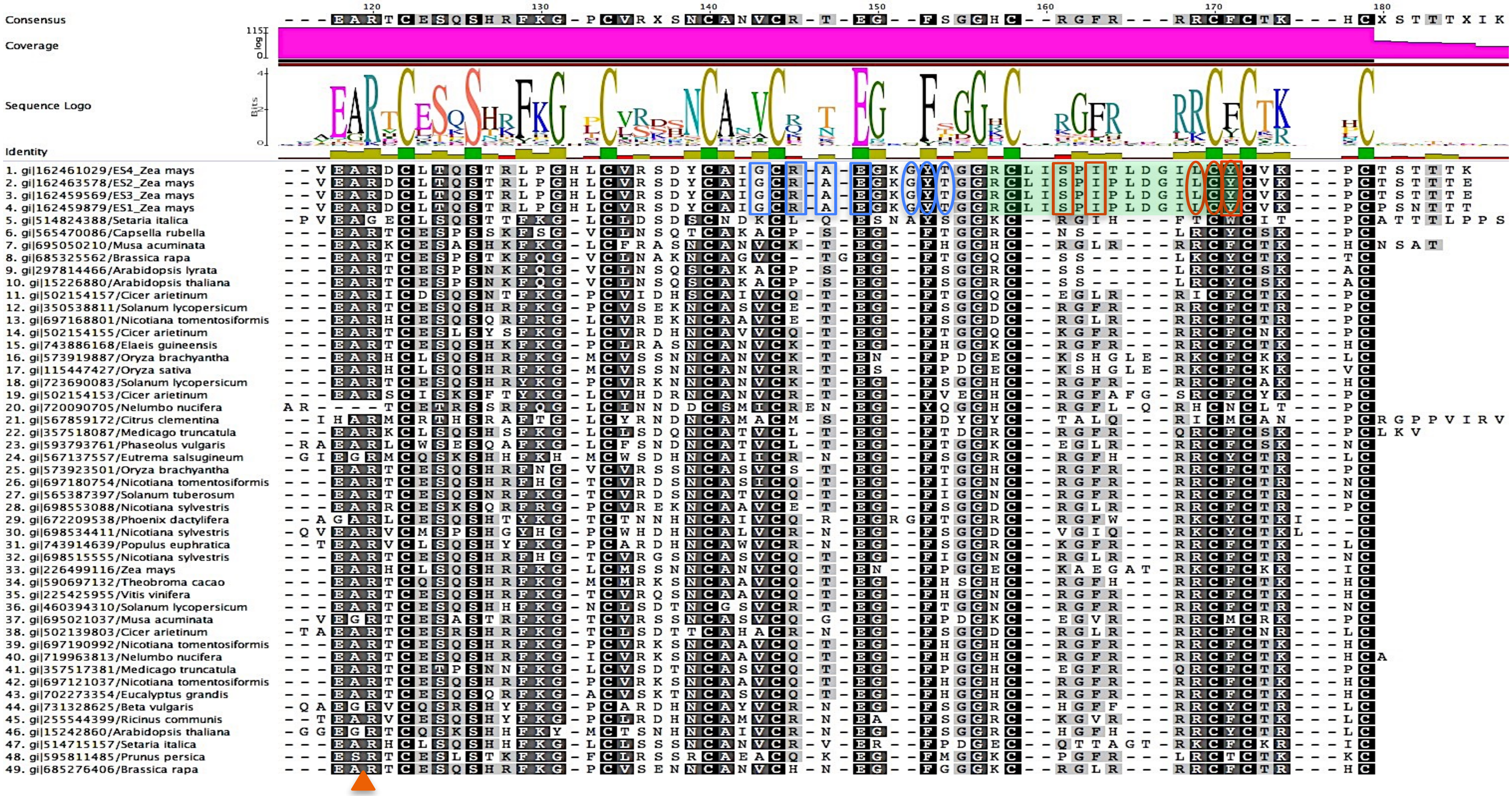

Figure S5. ES family peptides lack homology to plant defensins/DEFLs in regions responsible for their pollen tube burst activity. Maize ES4 was blasted against the refseq_protein database (Altschul et *a*l., 1997, 2005) and aligned with 115 homologous proteins found in 40 different species. All homologues belong to plant defensins and DEFL proteins (see also Table S1). Protein sequence alignment was exported and visualized using the Geneious software. The 49 proteins showing highest homology to ES4 are shown above. Maize ES1-4 are listed on top of the alignment. Amino acids are highlighted from light gray to black based on weak to high conservation. Amino acids of the DEFL motif (Knottin containing a typical pattern of eight cysteins) and the N-terminal signal peptide cleavage sites (indicated with an orange arrowhead) are highly conserved. The ES-d containing region (boxed in green), which mediates maize pollen tube burst, is highly polymorphic and different to other defensins and DEFLs. Furthermore, amino acids targeting maize pollen tube burst also show specificity to the maize ES family compared with amino acids targeting fungal growth inhibition. The most important amino acids affecting maize pollen tube burst (see also Figure 2C and D) are highlighted by blue and red boxes. Blue and red ellipsoids refer to amino acids affecting inhibition of fungal growth (see also Figures S2 and S3).

Supplemental references

Altschul SF, Madden TL, Schäffer AA, Zhang J, Zhang Z, Miller W, Lipma DJ. 1997. Gapped BLAST and PSI-BLAST: a new generation of protein database search programs. Nucleic Acids Res 25, 3389-3402.

Altschul SF, Wootton JC, Gertz EM, Agarwala R, Morgulis A, Schäffer AA, Yu Y-K. 2005. Protein database searches using compositionally adjusted substitution matrices. FEBS J 272, 5101-5109.
